# Challenges and experience of the Ethiopian rural health extension program: implications for reform and revitalization

**DOI:** 10.1186/s12913-023-10253-9

**Published:** 2023-11-27

**Authors:** Taddese Alemu Zerfu, Amare Abera Tareke, Sibhatu Biadgilign

**Affiliations:** 1 International Food Policy Research Institute (IFPRI), Addis Ababa, Ethiopia; 2 College of Medicine and Health Sciences, Dilla University, Dilla, Ethiopia; 3 Department of Biomedical Sciences, College of Medicine and Health Sciences, Wollo University, P.O.Box 1149, Dessie, Ethiopia; 4 Department of Public Health, College of Medicine and Health Sciences, Wollo University, Dessie, Ethiopia; 5 Independent Public Health Analyst and Research Consultant, PO.BOX 24414, Addis Ababa, Ethiopia

**Keywords:** Health extension program, Challenges, Revitalization

## Abstract

**Background:**

Despite remarkable gains over the past decade, mounting evidence suggests that Ethiopia’s rural health extension program (HEP) is facing serious implementation challenges. We investigated the current and potential future program design and implementation challenges of Ethiopia’s rural HEP based on the lived experiences of health extension workers (HEW) implementing the program at the grassroots level.

**Methods:**

We employed a longitudinal qualitative exploration linked to a larger cluster-randomized trial (RCT) which was implemented in 282 villages randomly selected from 18 *Kebeles* of the Gedeo zone, southern Ethiopia. Data were collected using in-depth interviews with key informants, focus group discussion, and passive observation of program implementation. The data were analyzed manually using a thematic framework analysis approach. Themes and sub-themes were generated by condensing, summarizing, and synthesizing data collected in the field in the form of extended notes and field observation checklists.

**Findings:**

Despite considerable gains in availing basic health services to the rural population, HEP seems to suffer serious design and implementation flaws that demand thoughtful and immediate adjustment. The design constraints span from the number and type of intervention packages to the means of dissemination (vehicle) as well as the target population emphasized. As such, some low-cost high-impact interventions that were strongly desired by the community were overlooked, while others were inappropriately packed. The means of distribution - female health extension workers trained with basic prevention skills, were lacking essential skills. They also had high burnout rates and with little engagement with men, were repeatedly mentioned flaws of the program demanding revitalization. Furthermore, the sheer structure of HEP precluded adult and adolescent men, non-reproductive women, and the elderly.

**Conclusion:**

Despite significant gains over the last couple of months, Ethiopia’s rural HEP appears to have reached a tipping point that requires a comprehensive revamp of the program package, means of distribution, and target beneficiaries rather than the “usual” tweaks to reap maximum benefits.

**Supplementary Information:**

The online version contains supplementary material available at 10.1186/s12913-023-10253-9.

## Introduction

Community health workers (CHWs) have contributed immensely to the expansion of access to healthcare for vulnerable populations around the world, such as those who have historically been marginalized and communities living in remote locations [1, 2]. They are typically regarded as a crucial component of the health workforce to address unmet health needs, increase access to services, deal with health inequities and enhance the effectiveness and efficiency of the health system [2].

With the premises of accelerating the country’s progress in meeting health related Millennium Development Goals (MDG) in 2003, the government of Ethiopia launched an innovative community based health extension program (HEP) as a primary component of the health sector development program (HSDP) [3, 4]. HEP is not a single or a specified group of curative interventions; rather “a package” of basic and essential promotive, preventive and few selected high impact curative health services. The underlying philosophy of HEP is the fact that if the right knowledge and skills is transferred to households, then they can take responsibility for producing and maintaining their own health and society at large [5, 6]. HEP has 18 packages of intervention geared toward the attainment of the highest health at the grass root level (Table 1).

**Table 1:** Selected HEP interventions, Federal ministry of health, Ethiopia

Maternal and child health • Antenatal care • Tetanus toxoid vaccinations • Iron folate supplementation • Family planning: oral contraceptive pills (OCP), condoms, injectable and implants	Diarrheal disease management (oral rehydration salt (ORS) and Zinc)
Disease prevention and control (curative services) • Human immunodeficiency virus (HIV) testing and counselling • HIV testing and counselling • TB prevention and control • Directly observed treatment short course therapy (DOTS) • Malaria prevention and control • Insecticide treated bed nets (ITN) • Indoor residual spray (IRS) • Malaria treatment	Pneumonia treatment (i.e., cotrimoxazole, amoxicillin, gentamycin
Hygiene and environmental sanitation • Improved water source • Hand washing with soup • Hygienic disposal of children’s stool • Latrine use	Expanded program of immunization (EPI) • Pentavalent vaccination • Measles vaccination • Pneumococcal vaccination

Since its rollout, HEP resulted in substantial improvements in areas related to disease control and prevention, family health, hygiene, and environmental sanitation. It has been the principal vehicle in expanding access to essential health services packages to all Ethiopians, with specific focus on women and children as the central piece [4, 7]. The program has also made a significant contribution to Ethiopia’s achievement of MDG 4 ahead of the 2015 timeline by reducing under-five death rates by 67%. [8–10].

However, despite considerable achievements over the past couple of decades, HEP is facing critical challenges and limitations that demand closer attention to a paradigm shift or significant revisions in its package of intervention, means of distribution and target population served [11]. Studies have shown huge turnover rate [8, 12], deterioration of quality of services (e.g. repeated measles epidemic in spite of ‘high’ immunization coverage) [13, 14], limited technical skills to handle skilled care [15, 16], poor communication skills with the community [17], absence from work place post [17], less sensitiveness to cultural issues in health, lack of social mobilization skills [18], weakened social influences [18, 19] and networks and limited support from the leadership [12]. However, there is a paucity of evidence regarding alternate approaches and decomposed options regarding areas for improvement. Therefore, the present study aims to elucidate a deeper insight to the challenges and experience of the rural HEP. Many countries who have introduced CHWs are facing challenges in maintaining high effective coverage and quality [20]. As an early pioneer of the CHW model at scale, Ethiopia’s experience is of considerable relevance to other countries who are considering how to institutionalize and universalize CHW models.

## Methods

### Study setting

**Fig. 1:**
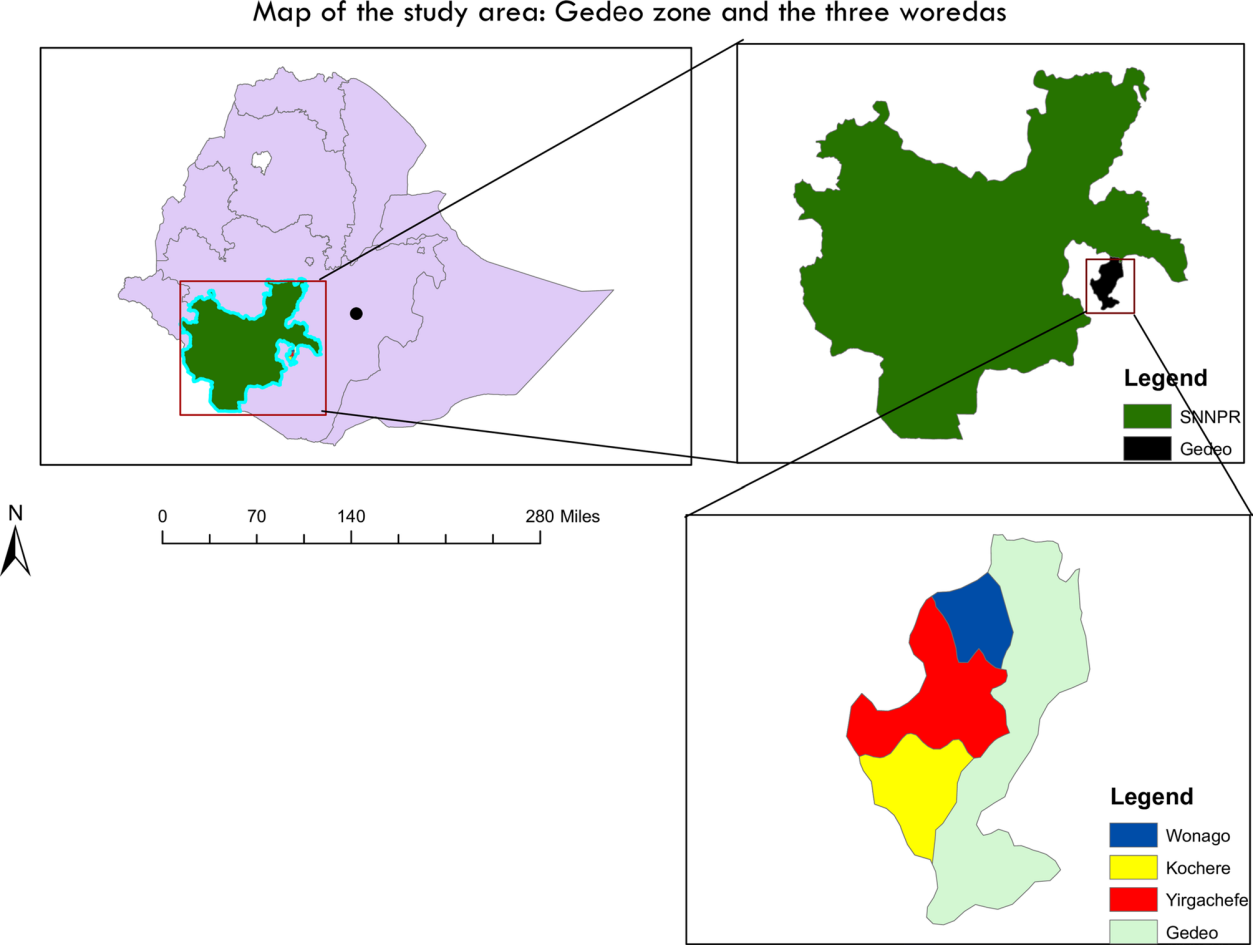
Map of the study area, showing the three woredas in Gedeo zone. SNNPR; South Nations Nationalities and Peoples Region

The study was conducted in 282 villages from 18 randomly selected *Kebeles* (smallest administrative units) in three districts of Gedeo Zone, Southern, Nations, Nationalities and Peoples (SNNP) region of Ethiopia. The three districts are: Wonago, Yirgachefe and Kochere [Fig. 1] having a total estimated population of 284,450, 320,012 and 187,654; respectively [21]. Dilla University Hospital is a referral center for the catchment population serving the largest population group in the area.

### Study design

This study is a continuation of a larger cluster randomized trial (RCT) that was implemented in Gedeo zone. Details on the process and implementation of this project were given in the trial registration website and reported elsewhere [21]. Briefly, it involved deployment of skilled and trained nurses called community based reproductive health nurses (CORN) to improve maternal health indicators including antenatal, skilled birth attendance (SBA) and delivery service. The implementation of the trial was conducted stepwise in four phases: preparatory, pre-implementation (training), implementation and termination (completion) phases. For qualitative study, we employed in-depth interviews and focus group discussions to collect comprehensive information throughout the project implementation period [22] between December 2014 and February 2016. Observations were conducted weekly using checklists.

### Study participants, sampling procedure and field data collection

Targets for this qualitative study were health extension workers (HEWs), district Health office representative, kebele administrators, pregnant and lactating mothers, family planning users, community leaders, husbands of pregnant or lactating mothers and elderly. Interviews and focus group discussions (FGDs) were conducted on these communities. The focus of the interview and FGDs were to identify and elucidate the key challenges and operational bottlenecks that the rural HEP is facing in the study area over time. The number of sessions (interviews or FGDs) were dependent on data saturation, hence, a total of 114 key informant in-depth interviews and 32 focus group discussions were held with the study participants alongside 164 observations. Interviews were conducted using a semi structured checklist [attached as supplementary file]. We conducted FGD in each of the Kebeles with mothers and husbands of index mothers aiming at identifying the limitations and constraints of HEP in their local context. Each of the FGD lasted for about 1 and 1/2 hour. The series of interviews and FGDs were compiled together.

### Data analysis

Data analysis was started in the field and built on existing findings. Data collected in the form of expanded notes and field observation checklists were condensed, summarized and synthesized to make thematic answers. These were later transferred to a manual thematic framework analysis table to facilitate coding and analysis. Each observation and expanded note were coded line-by-line to flag ideas and statements related to the study objective, and then codes were grouped to identify themes that were related to the factors that affected the implementation of HEP at grass root level. A priori themes were coded based on study objectives and emergent themes were identified based on the narratives of research participant. For better analysis and presentation of these challenges, we used the Lancet’s latest definition and organization of a strategy [23]. Accordingly, the Lancet describes a strategy as specification of the component intervention package, target group, and means of distribution.

### Ethical consideration

Dilla University College of Medicine and Public Health ethical review board approved the study. Written informed consent was sought from each participant before the interview began, and after explaining the purpose of the study. In case of illiterate participants, after explanation about the study, consent was taken by signature (fingerprint) along with a witness. The decision to participate in the study was not linked to the service participants were entitled to obtain. Written consent was archived at Dilla University College of Medicine and Public Health CORN project coordination office data management unit. All participants received a unique identification number that was used on the recorded interviews. Access to the raw data was limited only to key research team members. All the interviews were conducted by ensuring the privacy of the interviewee.

## Results

### Profile of study participants

A total of 114 key informant in-depth interviews, 32 focus group discussions and 162 systematic observations were held with the study participants. The observations were conducted both in the field (at community and household level) and at service provision sites (health posts). The mean age of study participants was 27.6 years, ranging from 19 to 56 years. Details on study participants profile is given in Table 2.

**Table 2:** Socio-demographic characteristics of study participants

Socio-demographic characteristics		No (%)		
Sex				
Male		33 (22.6)		
Female		113 (77.4)		
Age (years)				
19–25		38 (26)		
26–34		44 (30.1)		
35–44		24 (16.4)		
45–54		22 (15)		
55+		18 (12.3)		
Educational status				
No formal education		30 (20.5)		
Read and write only		33 (22.6))		
Primary school		23 (15.7)		
Secondary school		31 (21.2)		
College education and above		29 (19.9)		
Role in the community				
HEW		13 (8.9)		
Other health worker		12 (8.2)		
Community leader		26 (17.8)		
Other community member		95 (65)		

### Overview

The rural HEP is one of the successful development strategies, that government of Ethiopia has been recognized for useful health outcomes obtained since the initiation. On the other hand, there are emerging and reemerging challenges and operational bottlenecks of the strategy demanding closer attention. The following is a component-by-component description of the limitations of HEP by component intervention package, target group, and means of distribution as reported by the participants and observed in the field:

### I. limitations related to component intervention packages

The rural HEP had 16 component interventions packages that rose to 18 after the revision **(**Table 1**).** These intervention packages were further grouped into maternal and child health, disease prevention and control, hygiene and environmental sanitation, curative services (pneumonia treatment), and expanded program of immunization (EPI).

The overall focus of the component intervention packages is health promotion and disease prevention, with little emphasis to curative services. Generally, in addition to the depth and breadth of service limitations presented above, the HEP was identified to have several systemic and design related limitations described below.

### a) Missed or poorly addressed interventions

In the present study, it was observed that, none of the HEWs were seen providing curative and rehabilitative services that have been strongly demanded by the rural and grass root communities. The community were seen to have huge unmet needs for some essential curative and rehabilitative health services.

*‘’ …. the HEWs are helping our community; we feel things have improved much from previous times…. we can easily get vaccination, family planning and other health education to the nearest health post… but our community is highly demanding simpler curative services like deworming, drug administration, wound treatment and skilled birth attendants like your new nurses who came here now [they are referring the CORNS] …. we travel long distances to get these simple services which could easily be rendered by the HEW. I kindly ask the government to keep on deploying such professionals in our kebele….‘’*.

Administrator of a *Kebele* in Wonago district

### b) Weak program level integration with other relevant sectors

The other key programmatic limitation identified was lack of clear and administrative integration and linkage with key sectors at grass root level, i.e., education, agriculture, and economic sectors. HEWs’ training and implementation program packages merely focused on the health packages, lacking on ways to integrate with the education or agriculture sector.

*“…. as part of our day-to-day activity, we often work with agricultural extension workers and schoolteachers on some common interests…. But we didn’t get formal training on how to work and why to work with them… we feel that we are forced to work by local authorities…. why do I worry about farming while leaving my weekly schedule health packages program that I am accountable for…”*.

Health extension worker from a rural kebele

The linkage of HEP with academic institutions (Universities, colleges, and institutes), research centers and higher-level health facilities was also reported to be weak or absent.

*“… even though the country witnessed global level recognition for designing and implementing the HEP, the linkage and integration with key patterns and supporters of the health system is weak… we don’t have linkages with universities or research centers in the country, … we are just implementing without progressive evaluations and research in the program… we miss this component….”*.

HEP coordinator in one the district

### II) Limitations related to means of service distribution

**a) Two female HEWs**.

There were several limitations that are arising in deploying two female HEWs per kebele. The first limitation is that the program hardly emphasizes maternity and other leaves (which is reported to be common in rural areas) of the female HEWs. On the other hand, given the dynamics of demand for health services and population growth, HEP not updating the staffing profile of the HEP.

Field observation and interviews held with HEW showed that population growth and expansion of *Kebeles* coupled with long service year has been the critical challenge to handle HEP activities by two female HEWs. Furthermore, given the highly rugged topography and geographic setups of the rural areas and poor access to all weather roads, female HEWs are facing serious challenge to reach all households, particularly during rainy seasons.

*“… Even if the community and mothers prefer female health extension worker to serve them, as there are several issues to discuss privately, we females are not best fits like men to accessing remote and highly rugged mountainous areas of Gedeo… I think men should be there with us…. they can also help us in other mobilization activities which they are better-off than a female HEW….”*.

Heath extension worker in one of the rural kebele

### b) HEP delivered through a door-to-door visit walking on foot

The HEP design assumed that HEW can provide all the service packages travelling on foot on daily basis. It was also designed in such a way that HEW live in one of the rooms of the health posts. Nevertheless, in contrarily to these assumptions, we observed that almost all the HEWs live far from the health post, in their own home, travelling each day on foot to come to the facility. They also travel long distances for outreach services and to get to health centers as well as district health offices for various job-related activities. This has been overtly reported as the main bottleneck that significantly affected the effectiveness of the program. It is considered as the main reason for untimely burnouts and job burden among HEW.

*“… In my experience working here with the HEW, … I see that the HEWs are quite tired, fed-up with the huge job burden and other limitations… they are often absent from the HP, most stay-at-home caring for their babies… they work only when there is a campaign or when they know the supervisor is coming…., otherwise; they are quite tired, they frequently report that walking long distances on foot is the major problem for them…”*.

CORN who served for nine months in one of the rural kebele

In the same way, health service managers, community leaders and kebele administrators believe that the working condition for HEW is so difficult and needs to be changed quickly.

*“…. we know that there are many limitations that affect the program but walking on foot is not comparable to the other challenges…. I’ve seen that a newly deployed HEW can only effectively work traveling on foot across all villages for the first year, after that… they get tired… the job is so difficult, how on earth a woman travels all day 15–20 kilometers on foot for long years, I don’t think this is possible for anyone.”*

Kebele leader in one of the rural areas

*“… walking fifteen to twenty-five kilometers a day is really a difficult task…. our HEW are expected to function in that way which is difficult for anyone as a human being… there should have been a means of transportation …. we even are ashamed to criticize them for some jobs left undone because we know the pain….”*.

HEP coordinator in a district

### c) Career structure & working condition for HEW

Despite a visible success of the HEP and significant potential contribution made by HEWs to the national health system, HEP is facing challenges in terms of contextual factors and health system context that need to be addressed. Study participants mentioned challenges including poor and inauspicious living condition, poor housing and unavailability of certain amenities, lack of suitable career structure, lack of reading materials, lack of conditions for salary and operational budget, unconducive working hours, administration, poor monitoring and supervision, and inefficient relationship with communities.

*… I have been here serving the community since the inception of the program. During the initial years, we had huge motivation mechanisms including repetitive capacity building trainings, and regular supervision. Nowadays, there is little of such things ….*

A married Health extension worker and mother

### III) Target population for HEP

Even though the HEP aspires to empower the entire family and assumes no prejudice and the knowledge and skills to sustain health, it gives a special emphasis to mothers and children under five. Of the 18 packages, almost two-thirds (11 out of 18) are non-specific and refer to the whole family, and 5 (20.4%) are directly tailored to mothers and children. However, there is no single intervention directly relating to the health needs of adult men, the elderly, or other population groups. As previously stated, the lack of men-targeted services, as well as the means of service distribution (service provider) being female, make it less sensitive and receptive to addressing adult males and others.

### Lack of male involvement

Observations during weekly supervisions and monthly field visits showed that HEW had little or no contact with adult men as well as the youth. In almost all their day-to-day activities, be it at the health post or in the community (households), HEWs were seen serving mothers and under five children, with very limited contact with the adult men or elderly.

Reviewing HEP foundation documents, implementation guidelines and training materials; we also learned that HEP targets entirely mothers and children. Involving men in health programs in general and reproductive health is a critical program design limitation. The HEP foundation document clearly states that the program is mainly focusing on females because of a firm belief that most of the HEP packages are to be related to issues affecting mothers and children; thus, communication was thought to be easier between mothers.

Furthermore, community’s culture, perceived acceptability, and psychological readiness of HEW has contributed for missing men and restricted the program in addressing their health needs.

*“…our program mainly focusses on mothers and children…we were trained to reduce maternal and child mortality and have little to do with adult men…. in our culture also, it is not socially acceptable that women have close contact with men…….”*.

Health extension worker

*“… almost all the services are focus on women, this was deliberate from the very design of the program. HEP has little to do with men, adolescent and elderly population. It must be redesigned addressing the needs of these population, too….”*

A district health extension supervisor

## Discussion

Like many countries, Ethiopia’s HEWs were introduced to target the high burden of maternal and child morbidity and mortality. As Ethiopia evolves, including through an epidemiological transition towards a higher burden of non-communicable diseases, the long-term design and role of its CHW program needs reconsideration. This is something that many countries are grappling with [24]. This paper provides important evidence on the challenges of sustaining a scaled CHW program and identifies areas that will need addressing in the ‘New Era’ of CHW programs.

We conducted a qualitative assessment using in-depth interviews of key informants, focus group discussions, and observation of day-to-day activities of the HEP in one of Ethiopia’s rural areas. We aimed to elucidate existing and potential implementation and program design challenges of the once “highly contemplated” innovative and exemplary community health service delivery approach unique to Ethiopia, the Ethiopian HEP. Synthesizing the results, we learned that the HEWs have done an astonishing job to improve the overall health of the society since the inception of the program. Ethiopia, as a nation has benefited hugely from the performance of these strong and hardworking community health workers.

Nonetheless, despite its astounding successes, currently the program is facing inauspicious challenges, ranging from the dimensions of integration and collaboration with other sectors, to logistics challenges, adequacy and correctness of the number and gender mix of service providers, as well as systematic design errors comparable to the country’s emerging health needs and health system dynamics.

The major component of the program, the means of service distribution or the HEWs, on top of avoiding some critical interventions targeting other population groups (like men and adolescents), they seem to lack the essential skills and capacity to deliver high impact maternal and newborn interventions such as SBA, long-acting family planning and essential neonatal care services. Likewise, it would be impossible to maintain the successes gained and trajectory into the Sustainable Development Goals (SDGs)achieving maternal and neonatal targets.

Another issue noted in the current assessment was the difficulty in maintaining HEWs’ quality of time spent on actual and direct service-related activities. Other studies also show that HEWs worked as few as 25 h a week in Ethiopia, with differences across regions, and these working hours were not focused on priority concerns [25]. A large amount of time was spent on client waiting, rarely spending their time on major activities [25]. Other studies showed that HEWs spend, on average, 50 min travelling to work and 7 h and 49 min at work each day [17]. Much of the work they performed were seasonal, driven by campaigns for specific health interventions and management for disease outbreak [26]. These factors lead the HEWs to divert their attention to these seasonal activities and miss or give poor priority to the essential maternal and child health services.

The Ethiopian HEP was also found to be weak in addressing health issues that are not covered by the outreach programs [27]. It also focused little on adult health. Given the fact that Ethiopia is under epidemiological and demographic transition, the burden of non-communicable diseases is rising that calls for an innovative and quality of care at grass root level. However, HEP has given little or no emphasis to these emerging and re-emerging health problems.

The other key programmatic limitation identified during the present evaluation is lack of clear integration and linkage with key sectors at grass root level, i.e., education, agriculture, nutrition/food systems, and the economic sectors. Other studies have documented that HEP had little support from higher officials in the zonal and regional administration [26]. One of the key challenges in the program was the administrative systems; inconsistent support from district health offices, partly due to competing priorities, such as the management of disease outbreaks; and infrequent supervision of HEWs at the grassroots level.

As indicated in our result and previous reports [28]while female health extension worker is good for the overall maternal health services, being female only, is not such suitable for the HEWs and male involved health care services. Some of the HEWs believe that their salary was not adequate and if there were male workers their current salary might have doubled. And the HEWs being by themselves (without male colleague) are concerned for their personal safety, mentioning the risk of sexual harassment [28]. These issues may jeopardize the good actions provided by them. Even maternal health is maintained when the chief decision maker in the household (the husband in Ethiopian context) is involved. [29]. Household-centered disease prevention and health promotion initiatives, on the other hand, are closely associated with the husband. Again, illness screening/intervention programs should involve adult males.

Like most of the Ethiopian women particularly in rural setting, the HEWs have heavy domestic and caring workloads at home. In addition to the services offered at the health post and community level, HEWs have a significant deal of responsibilities at home, including cooking, cleaning, fetching food, and collecting water, which adds to their labor. In their roles, many of them are overburdened due to the laborious nature of their job; walking long distances, carrying heavy equipment like scales and record-keeping folders; and, unpaid overtime and low pay [25]. Two HEWs are expected to overcome these challenges, despite the increased number of populations since the launching of the program. Besides, the HEWs are expected to accommodate new campaigns, new additions to the package and work with other sectors like agriculture and education.

Transportation by foot consumes considerable amount of HEWs time and energy daily. Other studies have shown that closer to 10% of their time was consumed by travel [17], this will increase in door to door activities and seasonal campaigns. Lack of transport impacts on HEWs’ ability to reach the locations they are meant to serve, limiting their ability to support women needing to travel to access health services [28]. Besides the time and energy, it poses greater dissatisfaction and misery in their life. This finding is consistent with a study conducted in Illubabora Zone; South West Ethiopia suggested that providing transportation is another important mechanism to reduce the workload [30]. Similar finding was observed in other studies in which 41% of HEWs’ was spent on travel in 2014 study [17] and a study in 2017, out of the total observed work time, HEWs spent 16% of their time through travel between work activities [25]. Likewise, about 16% of their time HEWs’ time each week was spent for curative health activities [17, 32]. Maternal and newborn health services delivery were affected by community-related barriers such as distance, topography [31]. Similar to our finding, the living conditions and their working situations affect the utilization and quality of services offered by the HEWs [32] as well as living and working conditions of HEWs were not conducive during the early phase of the implementation of the HEP [5, 32], hence, HEWs were deployed in remote areas where housing was very important in motivating and retaining mechanisms in the communities leads to burnout or emotional difficulties.

Furthermore, the magnitude of HEWs ' intention to leave their current position was high due to a heavy workload, a lack of enthusiasm, and a limited career structure, which were all significant predictors of turnover intention. [30]. Workload, large distances, and a lack of support were all highlighted as problems that could demotivate HEWs from providing outreach services. The HEWs were obliged to execute different sectors’ tasks other than those in the health sectors; as a result, there would be an elevated workload on the existing health extension worker [30].

The other source of concern is, despite community needs, the lack of concentration or focus on providing curative and delivery services. District level experts and officials proposed staffing health posts with nurses to boost the quality of curative services [33]. Similarly to our findings, the prior study of community perceptions on HEP revealed that, with the exception of curative treatment and delivery services, there were very few concerns raised by the community. [6, 34].

## Conclusion and implications

HEWs have done tremendous job to improve child and maternal health by improving coverage of high impact interventions such as vaccination, ANC, institutional delivery and post-natal care, and prevention and control of infectious diseases. However, they lack the skills and capacity to deliver other low-cost, but high impact maternal and newborn interventions implementable at grass root level, such as SBA and neonatal care. Achieving maternal and neonatal targets will be difficult during the SDG era with the existing HEW or HEP approach. The HEP also face several challenges such as integration and collaboration with other sectors and logistical and transport challenges to achieve the existing packages. It is also noticeable not addressing adult men’s health into the sphere of spectrum in the design of the program. Cognizant of the above facts and to ensure long-term viability and benefit to those in need, the HEP should be revitalized such that the packages of services, the means of distribution, and the target population served are all tailored to the community’s needs.

### Electronic supplementary material

Below is the link to the electronic supplementary material.

Supplementary Material 1

## Data Availability

All data generated or analyzed during this study are included in this published article.

## References

[1] WHO. What do we know about community health workers? A systematic review of existing reviews. 2020. 10.1186/s12960-018-0304-x PMC6097220 30115074

[2] Swider SM (2002). Outcome effectiveness of community health workers: an integrative literature review. Public Health Nurs.

[3] Mohan PC. Ethiopia Health Sector Development Program. Africa Region Findings & Good Practice Infobriefs; No. 141. World Bank, Washington, DC. 2007.

[4] FMOH. Health extension program in Ethiopia - A profile. Addis Ababa. 2007.

[5] Kitaw Y, Ye-Ebiyo Y, Said A, Desta H, Teklehaimanot A. Assessment of the training of the first intake of health extension workers. Ethiop J Health Dev. 2007;21.

[6] Teklehaimanot A, Kitaw Y, Girma S, Seyoum A, Desta H, Ye-Ebiyo Y. Study of the working conditions of health extension workers in Ethiopia. Ethiop J Health Dev. 2007;21.

[7] Workie NW, Ramana GN (2013). The Health Extension Program in Ethiopia.

[8] Ethiopian Federal Ministry of Health. Health Sector Transformation Plan. (2015/16-2019/20). Addis Ababa, Ethiopia; 2015.

[9] Accorsi S. Special session “Countdown to 2015 in Ethiopia” Countdown to 2015: challenges and perspectives in achieving the Millennium Development Goals in Ethiopia. Articles from the 13th World Congress on Public Health. 2013;:7–12.

[10] Assefa Y, Gelaw YA, Hill PS, Taye BW, Van Damme W. Community health extension program of Ethiopia, 2003–2018: successes and challenges toward universal coverage for primary healthcare services. Global Health. 2019;15(1):1–11. 10.1186/s12992-019-0470-1 PMC6434624 30914055

[11] Okwaraji YB, Hill Z, Defar A, Berhanu D, Wolassa D, Persson LÃ (2020). Implementation of the ‘optimizing the health extension program ‘intervention in Ethiopia: A process evaluation using mixed methods. Int J Environ Res Public Health.

[12] University C for NHD of EC. Health Extension Program Evaluation: Rural Ethiopia Part - II. 2010.

[13] TEFERI A. Functioning of health extension program with particular focus on client-provider interaction in Ghimbo woreda of kaffa zone, SNNPR. Addis Ababa University; 2002.

[14] Herriman R. Ethiopia reports measles and diphtheria outbreaks _Outbreak News Today. 2015. Available at https://outbreaknewstoday.com/ethiopia-reports-measles-and-diphtheria-outbreaks-88130/#:~:text=by-,ROBERT%20HERRIMAN,-March%2029%2C%202015

[15] Medhanyie A, Spigt M, Kifle Y, Schaay N, Sanders D, Blanco R (2012). The role of health extension workers in improving utilization of maternal health services in rural areas in Ethiopia: a cross sectional study. BMC Health Serv Res.

[16] Karim AM, Admassu K, Schellenberg J, Alemu H, Getachew N, Ameha A et al. Effect of Ethiopia’s Health Extension Program on maternal and Newborn Health Care practices in 101 rural districts: a dose-response study. PLoS ONE. 2013;8. 10.1371/journal.pone.0065160 PMC3672192 23750240

[17] Mangham-Jefferies L, Mathewos B, Russell J, Bekele A (2014). How do health extension workers in Ethiopia allocate their time?. Hum Resour Health.

[18] Birhanu Z, Godesso A, Kebede Y, Gerbaba M (2013). Mothers’ experiences and satisfactions with health extension program in Jimma Zone, Ethiopia: a cross sectional study. BMC Health Serv Res.

[19] Sebastian MS, Lemma H (2010). Efficiency of the health extension programme in Tigray, Ethiopia: a data envelopment analysis. BMC Int Health Hum Rights.

[20] Schneider H, Olivier J, Orgill M, Brady L, Whyle E, Zulu J (2022). The multiple lenses on the Community Health System: implications for policy, practice and research. Int J Health Policy Manag.

[21] Zerfu TA, Taddese H, Nigatu T, Tenkolu G, Vogel JP, Khan-Neelofur D (2017). Reaching the unreached through trained and skilled birth attendants in Ethiopia: a cluster randomized controlled trial study protocol. BMC Health Serv Res.

[22] Maxwell Ja. Designing a Qualitative Study. The SAGE Handbook of Applied Social Research Methods. 2009;:214–53.

[23] Campbell OM, Graham WJ (2006). Group LMSS steering. Strategies for reducing maternal mortality: getting on with what works. The Lancet.

[24] Zulu JM, Perry HB (2021). Community health workers at the dawn of a new era. Health Res Policy Syst.

[25] Tilahun H, Fekadu B, Abdisa H, Canavan M, Linnander E, Bradley EH (2017). Ethiopia’s health extension workers use of work time on duty: time and motion study. Health Policy Plann.

[26] Okwaraji YB, Hill Z, Defar A, Berhanu D, Wolassa D, Persson LÃ (2020). Implementation of the ‘Optimising the Health Extension Program’ intervention in Ethiopia: a process evaluation using mixed methods. Int J Environ Res Public Health.

[27] Karim AM, Admassu K, Schellenberg J, Alemu H, Getachew N, Ameha A (2013). Effect of Ethiopia’s health extension program on maternal and newborn health care practices in 101 rural districts: a dose-response study. PLoS ONE.

[28] Jackson R, Kilsby D, Hailemariam A (2019). Gender exploitative and gender transformative aspects of employing Health Extension Workers under Ethiopia’s Health Extension Program. Tropical Med Int Health.

[29] Emaway Altaye D, Karim AM, Betemariam W, Fesseha Zemichael N, Shigute T, Scheelbeek P. Effects of family conversation on health care practices in Ethiopia: a propensity score matched analysis. BMC Pregnancy Childbirth. 2018;18 Suppl 1:372. 10.1186/s12884-018-1978-8 PMC6157286 30255781

[30] Kitila KM, Wodajo DA, Debela TF, Ereso BM (2021). Turnover intention and its Associated factors among Health Extension workers in Illubabora Zone, South West Ethiopia. J Multidisciplinary Healthc.

[31] Higi AH, Debelew GT, Dadi LS (2021). Perception and experience of Health Extension workers on facilitators and Barriers to Maternal and Newborn Health Service Utilization in Ethiopia: a qualitative study. Int J Environ Res Public Health.

[32] Assefa Y, Gelaw YA, Hill PS, Taye BW, Van Damme W (2019). Community health extension program of Ethiopia, 2003–2018: successes and challenges toward universal coverage for primary healthcare services. Globalization and Health.

[33] Defar A, Alemu K, Tigabu Z, Persson LÃ, Okwaraji YB (2021). Caregivers’ and Health Extension workers’ perceptions and experiences of Outreach Management of Childhood illnesses in Ethiopia: a qualitative study. Int J Environ Res Public Health.

[34] Bekele A, Kefale M, Tadesse M. Preliminary assessment of the implementation of the health services extension program: the case of southern Ethiopia. Ethiop J Health Dev. 2008;22.

