# Supplementary material for: Challenges and experience of the Ethiopian rural health extension program: implications for reform and revitalization
Source: BMC Health Serv Res. 2023 Nov 27;23:1309. doi: 10.1186/s12913-023-10253-9 (PMC10683286; doi:10.1186/s12913-023-10253-9)
Supplement: Supplementary file 1 — Supplementary Material 1 [file 12913_2023_10253_MOESM1_ESM.docx]

**KII guide with CORNs and Health officials and experts**

1. Please tell us about the situation of HEP implementation in the area.
   - Level of acceptance and use of the HEP by the community?
   - Degree of involvement and support?
   - Which of activities are most executed, and which ones not? Why?
   - Which activities are more challenging? Why?
2. Tell us about the means of service provision (the HEW)?
   - How motivated are the HEW?
   - What is/are the common services they provide?
   - How often and frequent is their visit?
3. If HEW are facing any challenge, tell us more about this?
   - Are the HEW happy about their work? What is the most frustrating thing in their day-to-day activity?
   - What challenges they encounter?
     - Operational challenges?
     - Self-development and productivity?
     - Inputs and service delivery materials?
4. Tell us about how the community perceives the HEP?
   - - Which of the services they use most?
     - Which services have less demanded or used least? Why?
     - What additional service needs do you have?
5. Tell us about the other factors that influence the use of HEP in the area?
   - Knowledge and attitude about the benefit of HEP in general and service providers, i.e., HEW?
   - Workload of HEW?
   - Service uptake by the community?
   - Other challenges etc.
6. Tell us about how to strengthen the HEP?
   - Any revisions / corrections needed in the program?
   - Changes, if needed, to the number and mix of HEW?
   - Changes required in the number and mix of services?
   - Any other recommendations?
